# Novel *ARG1* variants identified in a patient with arginase 1 deficiency

**DOI:** 10.1038/s41439-021-00139-9

**Published:** 2021-02-04

**Authors:** Katsuyuki Yokoi, Yoko Nakajima, Toshihiro Yasui, Makoto Yoshino, Tetsushi Yoshikawa, Hiroki Kurahashi, Tetsuya Ito

**Affiliations:** 1 Department of Pediatrics, Fujita Health University School of Medicine, Toyoake, Japan; 2 Division of Molecular Genetics, Institute for Comprehensive Medical Science, Fujita Health University, Toyoake, Japan; 3 Department of Pediatric Surgery, Fujita Health University, Toyoake, Japan; 4 Division of Gene Therapy and Regenerative Medicine, Cognitive and Molecular Research Institute for Brain Diseases, Kurume University, Kurume, Japan

**Keywords:** Mutation, Molecular biology

## Abstract

We report a case of a 13-year**-**old boy with arginase 1 deficiency carrying a new variant in *ARG1*. Sanger sequencing identified the compound heterozygous variants: NM_000045.4: c.365G>A (p.Trp122*)/c.820G>A (p.Asp274Asn). Although not previously reported, the p.Asp274Asn variant is predicted to have strong pathogenicity because it is located in a highly conserved domain in the protein core and arginase activity in the patient was below measurement sensitivity.

Hyperargininemia is an extremely rare autosomal recessive condition that is associated with urea cycle disorder (UCD); it is caused by a deficiency of arginase 1, which hydrolyzes arginine to form ornithine and urea. *ARG1*, the gene encoding arginase 1, is located on chromosome 6q23, encompassing a 15-kb genomic region with 8 exons^1^. Patients with arginase 1 deficiency show high levels of plasma arginine, relatively mild or moderate hyperammonemia manifesting as spastic paraparesis, and progressive neurological symptoms^2^. The management of individuals with arginase deficiency should closely mirror what is described in Urea Cycle Disorders Overview (restriction of dietary protein and administration of oral nitrogen-scavenging drugs). The goal should be to maintain the plasma arginine concentration to as near the normal value as possible (normal range, 54–134 nmol/mL)^3^. The incidence of arginase 1 deficiency has been reported to range between 1:300,000 and 1:2,000,000 live births^2^. The incidence of arginase 1 deficiency based on the combined database for the United States and Europe is 1:950,000^4^; of the six main UCDs, it is probably the third rarest, after NAGS (<1:2,000,000) and CPS1 (1:1,300,000) deficiencies^4^. In Japan, the incidence of arginase 1 deficiency may be less than that in the United States and Europe, i.e., an incidence of 1:2,200,000, per a previous report^5^. Here, we present a case of 13-year-old boy with hyperargininemia. A novel *ARG1* variant was identified in this patient.

A 13-year-old boy with arginase 1 deficiency who was born to parents with no consanguinity was assessed. His gestational age was 36 w 6 d, his birth weight was 3160 g, and he had no perigestational problems. He exhibited mild neonatal jaundice after birth, which improved after only one day of treatment of phototherapy on the 4th day of life. He was discharged from the maternity clinic on day 6. He showed normal growth afterward; however, at 30 days of age, a routine checkup revealed a hepaplastin test (HPT) value of 26%, which is low. Vitamin K syrup was administered, but he was subsequently admitted to the same hospital at 43 days of age for close observation because the low HPT value persisted. Upon hospitalization, physical examination showed no abnormality. The laboratory test results revealed the following: AST 72 U/L; ALT 45 U/L; TB 4.5 mg/dL; DB 1.6 mg/dL; ALP 1870 U/L; and HPT 20%. Coagulation element levels were not abnormal. Hepatic scintigraphy was normal. However, his serum amino acid levels at 51 days of age showed an extremely high level of arginine (1402.2 nmol/mL, normal range; 53.6–133.6) and hyperammonemia, with an ammonia level of 301 µg/dL. Breast and formula milk were stopped, and administration of protein-free milk, an essential amino acid mixture, and sodium benzoate was started. After these treatments, his hyperammonemia resolved, and the low level of HPT was improved at approximately 1 week (59 days of life: NH_3_ 59 µg/dL; arginine 129.2 nmol/mL; HPT 79%; AST 75 U/L; ALT 140 U/L; TB 1.2 mg/dL; DB 0.9 mg/dL; and ALP 1910 U/L). At the age of 3 months, he was referred to our hospital for treatment and follow-up of hyperargininemia. His protein intake was maintained at approximately 0.8 g/kg/d, which is indicated in the literature^6^ as the therapeutic dose for ARG1-deficient patients. Arginase activity in red blood cell extracts was measured in the same manner as in a previous report^7^, though no activity was detected (mother: 702; control: 1,894 µmol/hour/g hemoglobin). Therefore, we biochemically diagnosed him with arginase 1 deficiency because of the low enzyme activity. We continued protein restriction (0.7–0.8 g/kg/d) and administration of oral nitrogen-scavenging drugs. However, because serum arginine levels remained very high (500–800 nmol/mL), living-donor liver transplantation was performed at the age of 1 year and 5 months. His arginine level decreased immediately after the operation and remained within the normal range without a protein-restricted diet. He showed proper development without any hyperammonemia or neurological symptoms. His IQ was 90 by the WISC-III at 6 years of age, and he has attended school normally to date (current age: 12 years). Although his condition was biochemically diagnosed based on enzyme activity, genetic tests were performed to confirm the diagnosis. Genetic counseling was suggested, but the parents had no wish of a future pregnancy.

This study was performed in accordance with the Declaration of Helsinki. The study protocol was approved by the Ethical Review Board for Human Genome Studies at Fujita Health University. Written informed consent to publish the medical information was obtained from the patient and his parents. DNA was extracted from peripheral blood samples and sequenced using the Sanger method to screen for genetic variations at the nucleotide level in all coding exons of *ARG1*. We used the UCSC genome browser (http://genome-asia.ucsc.edu/human GRCh38/hg38) for human genome assembly. *In silico* analysis was performed using PolyPhen-2 and SIFT. Protein structure predictions were performed using PyMol (https://pymol.org/2/).

Genetic analysis revealed a compound heterozygous state, with NM_000045.4: c.365G>A (p.Trp122*)/c.820G>A (p.Asp274Asn; Fig. 1). Parental samples were also analyzed to detect the origin of the variants. The result showed that p.Trp122* and p.Asp274Asn were inherited from his mother and father, respectively. The c.365G>A mutation has been reported to be a pathogenic variant, but the c.820G>A variant has not yet been reported. Analysis of the c.820G>A substitution using SIFT and PolyPhen-2 prediction software indicated that this mutation is “damaging” and “probably damaging,” with scores of 0 and 1.000, respectively. The Asp274 residue is located in the protein core, and no collision between atoms that could affect the 3-D structure is predicted for the Asp274Asn mutation (Fig. 2A). Nevertheless, it is highly conserved among various species (e.g., human, rhesus monkey, mouse, dog, elephant, chicken, *Xenopus tropicalis*, and zebrafish; Fig. 2B). When this variant was evaluated using the American College of Medical Genetics (ACMG) recommendations, PM2, PM3, PP3, and PP4 were applied, indicating that it is “likely pathogenic”.

**Fig. 1:**
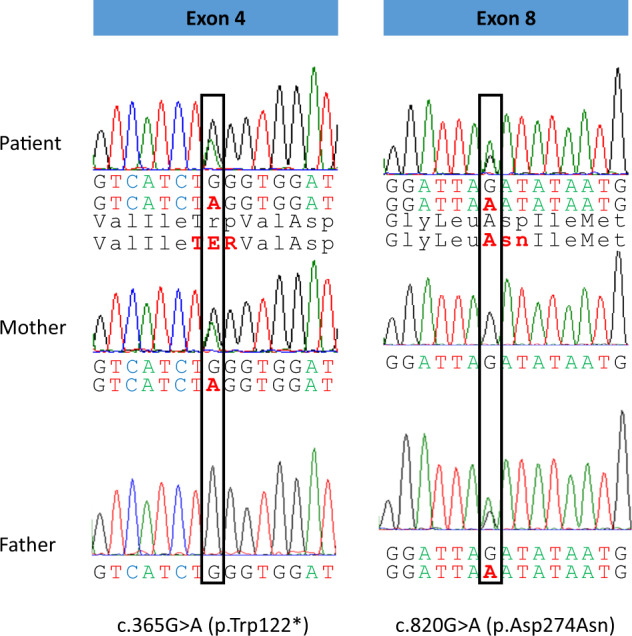
Sanger sequencing results for the *ARG1* gene in the patient and his parents and predicted amino acid sequences. Electropherograms of Sanger sequencing results. c.365G>A (p.Trp122*) in the patient and his mother are shown. c.820G>A (p.Asp274Asn) in the patient and his father is shown.

**Fig. 2:**
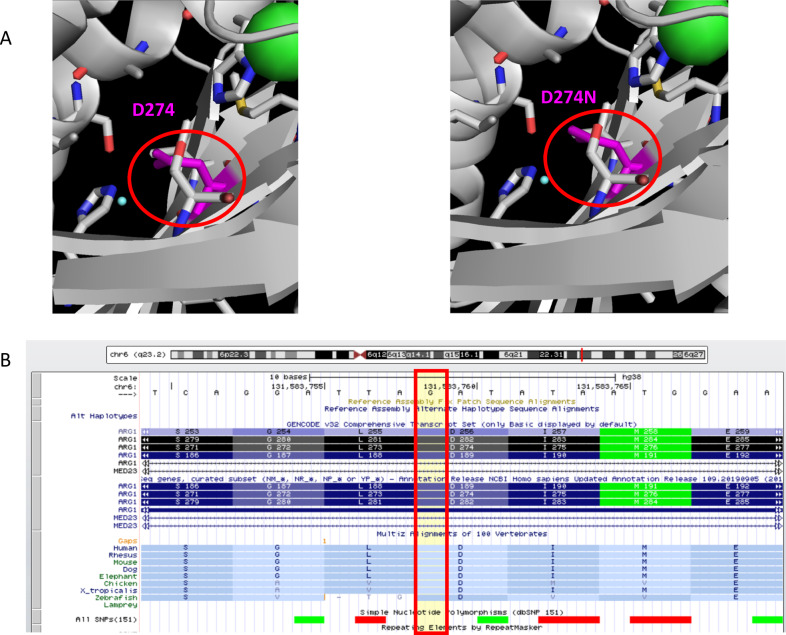
Prediction of arginase 1 structure. **A** Prediction of arginase 1 structure using PDB ID: 2AEB. Asp274Asn is located inside the protein. No collision between atoms that could affect the maintenance of the 3-D structure is predicted for the Asp274Asn mutation (purple). **B** Levels of conservation of the p. Asp274 domain in *ARG1* among different species are shown.

In this study, we identified a novel missense variant (p.Asp274Asn) in *ARG1* in a patient with arginase 1 deficiency. This variant is not listed in the database (gnomAD browser beta; http://gnomad.broadinstitute.org/) for the normal population. Structural analysis of the protein did not reveal any collisions between residues, but it is highly conserved, and enzyme activity in this patient was below the sensitivity of the measurement. Therefore, this variant is believed to have a significant effect on the protein structure of arginase. In this variant, the acidic amino acid aspartic acid replaces the neutral amino acid asparagine located in the protein core. It has been reported that the amino acids buried inside the protein are involved in protein structure^8^. Therefore, it has been considered that a change in the charge inside the protein might have some influence on its structure and function, even if atomic collisions are not predicted.

Arginase 1 deficiency is assessable in newborn screening through tandem mass spectrometry. However, the deficiency in patients might be missed if the screening occurs too early, as L-arginine levels may not be markedly elevated in the first days of life^9^. This may be the reason why arginase 1 deficiency has not been introduced as a target disease in Japanese newborn screening. This patient was diagnosed early because of the abnormal value obtained in the hepaplastin test. There has been no previous report of hyperargininemia patients with abnormal hepaplastin test values during the neonatal period. This patient’s severe symptoms were considered to result from the fact that one variant is a nonsense variant and the other is located in a highly conserved domain, with enzyme activity being below measurement sensitivity. Patients with severe hyperargininemia may have an outlier hepaplastin test result during the neonatal period.

## Data Availability

The relevant data from this Data Report are hosted at the Human Genome Variation Database at 10.6084/m9.figshare.hgv.2969.
